# Autism in Early Childhood: An Unusual Developmental Course—Three Case Reports

**DOI:** 10.1155/2012/946109

**Published:** 2012-06-17

**Authors:** Michal Cohen-Ophir, Tsophia Castel-Deutsh, Emanuel Tirosh

**Affiliations:** The Hannah Khoushy Child Development Center, Bnai Zion Medical Center and The Rappaport Family Faculty of Medicine, The Technion-Israel Institute of Technology, P.O. Box 4940, 25148 Haifa, Israel

## Abstract

Autistic spectrum disorder (ASD) is typically characterized by either an emerging and gradual course or developmental regression in early childhood. The versatile clinical course is progressively acknowledged in recent years. Children with developmental disorders in general are referred to the Child Development Center for a multidisciplinary assessment, investigation, treatment and followup. We report three infants with an initial diagnosis of developmental delays, recovery of normal development following intervention in a multidisciplinary center, and subsequent regression into classic autism following their discharge from the program. An extensive medical workup was noncontributory. This unusual presentation, to our knowledge not reported previously, should be recognized by professionals involved in child development and psychiatry.

## 1. Introduction

Autism is an heterogeneous developmental disorder mainly characterized by three domains of impairments: (1) communication/language; (2) social interaction; (3) behavioral oddities (DSM-4) [1]. Diagnosis in infancy is crather difficult before the fully developed, typical symptomatology develops [2]. In addition, the frequently associated mental retardation may also contribute to the delayed diagnosis [3]. Two predominant clinical courses were documented in the literature pertaining to autism in childhood. (1) The gradual course in which a typical behavior and development appears during the first two years of life. Parents sometimes recall certain symptoms earlier in the course of the second and occasionally the first year of life [4]. (2) The regressive course in which parents notice loss of developmental skills following normal or abnormal development, until the second year of life [5]. This paper was based on the analysis of parents' interviews and questionnaires, and therefore a recall bias might be expected [5].

Following the regression, very few regain normal development [6]. A proportion (41%) of the children who were reported to have loss of skills were also reported to have elevated rate of symptoms at an early age (10–12 ms). However, previous research also reported that children who were later diagnosed as ASD received the diagnosis of general developmental delay at an earlier age [7, 8]. 

The present paper includes three children who were diagnosed at the Child Development Center with various nonautistic developmental problems, who made a significant progress while being enrolled in a developmental program. They were discharged from the program following an assessment confirming their within normal developmental performance. The children were readmitted to the CDC following 7–24 months and diagnosed as having ASD. This clinical course to our knowledge has not been previously described in the literature pertaining to autism.

## 2. Methods

Three children who were assessed periodically by a multidisciplinary team at the Hannah Khoushy Child Development Center of the Bnai Zion Medical Center are described. This tertiary care center provides assessment and treatment for children with suspected developmental deficits from the Greater Haifa Area. The screening and referral procedure pertaining to child development in the area has been previously reported [9].

## 3. Procedure

Children who are suspected of a communication disorder are assessed for their communication and emotional behavioral aspects by the multidisciplinary team. The possible diagnosis of autistic spectrum disorders is routinely entertained and discussed. All suspected infants and children are subject to classification employing commonly used instruments.

### 3.1. Instruments

 For the diagnosis of ASD, an independent assessment of a developmental pediatrician and a psychologist and an agreed upon diagnosis of ASD are mandatory. The following instruments are routinely used: (1) Diagnostic and Statistical Manual IV (DSM IV) [1]; (2) the Childhood Autism Rating Scale (CARS) [10]; (3) developmental assessments employing commonly used inventories [11, 12]; (4) In children with a borderline diagnosis, the Autism, Diagnostic Interview Revised (ADI-R) is employed [13]. The three children included in the present paper were assigned the final diagnosis of ASD on the basis of the proposed criteria of these instruments. 

## 4. Results

### 4.1. Case Reports

**(1) O.S.**

A male infant, was assessed at the age of four months for a routine assessment of premature infants. He was a product of a 33-week uneventful pregnancy. BW was 2120 grams (appropriate for gestational age). Apgar score was 9/10. His physical examination revealed cleft lip. His family history was unremarkable. He was discharged home at the age of three weeks following phototherapy for physiologic jaundice (bilirubin 12 mg %). His assessment revealed cleft lip, a mild head tilt to the left, and mild hip adduction. His anthropometric data were at the 50th percentile. His neurological evaluation was concluded normal, and his developmental achievements were appropriate for corrected age in all domains. Auditory evoked brain potentials, and genetic consultation was unrevealing. His cleft lip was operated at the age of seven months. Followup assessment at the age of 13 months revealed normal physical and neurological examination, other then hyperlaxity of ligaments. Developmental assessment revealed normal social and language development, while his gross and fine motor development were scattered between eight and twelve months (he showed left-hand preference). His behavior was characterized by restlessness and difficult transitions that were easily consoled by his mother. He was found to have a mixed response to different sensory stimulations. A regulation disorder of mixed type was diagnosed [14]. He was treated for a period of 14 months by the developmental physiotherapist, and the family was consulted by the social worker for his behavioral-emotional vulnerability. He was discharged at the age of 18 months following the team's assessment, as a developmentally and behaviorally age appropriate infant, with no further parental concerns.

At the age of three years and seven months, the parents asked for reassessment. They reported language regression at the age of 21 months attributed to the birth of his sister. His physical and neurological assessments were unrevealing. Metabolic workup and EEG were found normal. Karyotype and DNA analysis for fragile-X were found normal. The diagnosis of autism was assigned. Repeated assessments until the age of five years confirmed the diagnosis. From the age of 3 years and eight months, he was treated at the special unit for children with ASD by a multidisciplinary team. His CARS score was 44 and ASQ score 25 (15 cutoff score).

**(2) ET.**

Male, was first referred at the age of two years and four months for attention and behavioral difficulties. He was the product of an eventful 39-week pregnancy with a BW of 2920 grams and Apgar score of 10/10. His family history was unremarkable. His medical history was unremarkable. However, he was noted to have expressive language delay and toe walking. His physical examination was unremarkable, head circumference and weight were of the 25th percentile and height at the 75th percentile.

Assessment was unremarkable but mild achilles tendon shortening bilaterally. His developmental assessment revealed a scattered profile in all areas between 21–28 months. His expressive language was determined at a level of 21 months. His nursery teacher reported a communication deficit associated with severe restlessness. He was treated by a social worker employing “dyadic therapy,” and parents were consulted weekly by a speech therapist. Hearing and vision as well as EEG were found normal. At the age of two years and 10 months, he was reassessed by the team, as a normally developing child with normal neurological examination, resolving habitual toe walking and mild temperamental difficulties and discharged from the center with no need for followup.

His language assessment revealed an age appropriate, one standard deviation below the mean of both expressive and receptive language. At the age of 41 months, the family asked for consultation at the Child Development Center, since they noticed stereotypic movements, poor mutual interest, and joint attention. His toe-walking reappeared, and immediate and delayed echolalia was emerging. Genetic workup was negative. He was assigned the diagnosis of ASD and referred to the center for ASD where the diagnosis was reconfirmed. His CARS score was 29.5.

**(3) IP.**

A 14-month-old male infant, was first referred with a history of congenital hypothyroidism and delayed general development. He has a healthy twin brother and a three-year and seven-month old sister treated by a speech therapist for delayed expressive language. He was a second born twin with a birth weight of 2200 grams (twin BW—3000 grams). He started Elthroxin treatment at three weeks of age. His physical and neurological evaluations were normal. His developmental assessment revealed a scattered performance between nine to twelve months. He appeared to have an under active temperament. TSH and T4 serum levels and hearing evaluation were normal. He was enrolled into a developmental program provided by a physical therapist. At 18 months, he was found to have normal gross, fine, and social development with delayed language. He showed typical joint attention. At the age of  38 months, he was reevaluated and autism was diagnosed based on DSM4 criteria and a CARS score of 48. Extensive metabolic workup, EEG, thyroid functions, MRI, and MRS were all concluded normal. He was referred to the special unit for the children with pervasive developmental disorders where the diagnosis of autism was reconfirmed. Reevaluations following six and 12 months of DIR/floor time treatment confirmed autism although some improvement in interpersonal communication was noted.

## 5. Discussion

To date, the main two clinical courses of ASD reported in the literature are the gradual course and the second is characterized by regression. Both types can be diagnosed in early or late infancy [15]. It has also been suggested that, among the children with late onset autism, about 65% would demonstrate an increased rate of suggestive symptoms during the first year of life [5]. Our present report pertains to a different course in which children regressed into ASD following a successful treatment for nonautistic developmental delay. A study [8] employing a prospective approach documented a developmental disruption among the later-diagnosed group (>14 month of age), but overall they were not found to have an ASD at the age of 14 months but rather a variety of other developmental problems. This later study did not account for the type of interventions employed with the children or their families throughout their followup. Although an extensive investigation including EEG, metabolic, and genetic workup as well as brain imaging was employed, no underlying pathology was revealed in any of the three children, reported herein. At the first stage, the children were assessed by a multidisciplinary team and developmental delay associated with emotional problems was confirmed and treated. Only following “normalization” of their developmental and emotional state and discontinuation of treatment, regression into ASD occurred. We are not aware of such clinical observation reported in the literature to date. The lack of any specific signal to the possible risk of future ASD of these children is alarming, yet it should be noted that parental retrospective interviews suggested regulatory behavioral problem among children who latter regressed into autism [15] similar to the course observed among the children included in the present paper.

 Previous uncontrolled observations suggested a significant clinical response to intervention using DIR/floor time in children with autism [16]. However, there is no account of a regressive course following a successful intervention. All three children were not fulfilling the diagnosis of ASD at their initial assessment. Whether continuation of developmental treatment could have prevented or postponed the age of ASD presentation is a matter of speculation. Following the diagnosis of ASD, all three children were enrolled into a program specifically designated for children with ASD. Neither was reported as recovering from this diagnosis, following six and 12 months of treatment.

## 6. Conclusion

The present paper portraits a unique course of a treated early nonautistic developmental delay with a recovery of typical development, subsequent to a developmental intervention and followed by an autistic regression, clinicians involved in child development should be aware of such course and consider a proper followup for children with developmental delay under their care.

## References

[1] (1994). *Psychiatric Association The Diagnostic and Statistical Manual of Psychiatric Disorders*.

[2] Stone WL, Cohen DJ, Volkmar FR (1997). Autism in infancy. *Autism and Pervasive Developmental Disorders*.

[3] Wing L (1997). Syndromes of autism and atypical development. *Autism and Pervasive Developmental Disorders*.

[4] Lord C (1995). Follow-up of two-year-olds referred for possible autism. *Journal of Child Psychology and Psychiatry and Allied Disciplines*.

[5] Werner E, Dawson G, Munson J, Osterling J (2005). Variation in early developmental course in autism and its relation with behavioral outcome at 3-4 years of age. *Journal of Autism and Developmental Disorders*.

[6] Rapin I, Katzman R (1998). Neurobiology of autism. *Annals of Neurology*.

[7] Cox A, Charman T, Baron-Cohen S (1999). Autism spectrum disorders at 20 and 42 months of age: stability of clinical and ADI-R diagnosis. *Journal of Child Psychology and Psychiatry and Allied Disciplines*.

[8] Landa RJ, Holman KC, Garrett-Mayer E (2007). Social and communication development in toddlers with early and later diagnosis of autism spectrum disorders. *Archives of General Psychiatry*.

[9] Tirosh E, Lechtman M, Diamand H, Jaffe M (1993). An effective community-based approach to the identification of neurodevelopmental delay in childhood. *Developmental Medicine and Child Neurology*.

[10] Schopler E, Reichler RJ (1988). *The Childhood Autism Rating Scale ( CARS )*.

[11] Gesell A, Amatruda CS (1954). *Developmental Diagnosis: Normal and Abnormal Child Development*.

[12] Griffiths R (1970). *Abilities of Young Children: A Comprehensive System with Mental Measurements for the First Eight Years of Life*.

[13] Lord C, Rutter M, Couteur AL (1994). Autism diagnostic interview-revised: a revised version of a diagnostic interview for caregivers of individuals with possible pervasive developmental disorders. *Journal of Autism and Developmental Disorders*.

[14] Egger HL, Fenichel E, Guedeney A, Wise BK, Wright HH (2005). *DC: 0-3R Diagnostic Classification of Mental Health and Developmental Disorders of Infancy and Early Childhood*.

[15] Werner E, Dawson G (2005). Validation of the phenomenon of autistic regression using home videotapes. *Archives of General Psychiatry*.

[16] Greenspan SI, Wieder S (2006). *Engaging Autism: Helping Children Relate, Communicate and Think with the DIR Floor Time Approach*.

